# Evaluating the Appropriateness and Feasibility of the Care Partner Hospital Assessment Tool (CHAT)

**DOI:** 10.3390/ijerph182413355

**Published:** 2021-12-18

**Authors:** Madeline Carbery, Richard Schulz, Juleen Rodakowski, Lauren Terhorst, Beth Fields

**Affiliations:** 1 Department of Kinesiology, University of Wisconsin-Madison, Madison, WI 53706, USA; mcarbery@wisc.edu; 2 Department of Psychology, University of Pittsburgh, Pittsburgh, PA 15260, USA; schulz@pitt.edu; 3 Department of Occupational Therapy, University of Pittsburgh, Pittsburgh, PA 15260, USA; jur17@pitt.edu (J.R.); lat15@pitt.edu (L.T.)

**Keywords:** caregiving, screening, hospital, aging, education, mixed methods

## Abstract

Hospital practitioners rely on care partners of older adults to provide complex care without identifying and addressing their needs. The Care Partner Hospital Assessment Tool (CHAT) was developed to identify the education skill training needs of care partners of hospitalized older adults. This two-phased mixed-method study evaluated the appropriateness and feasibility of the CHAT. The phase 1 quantitative survey with caregiving experts indicated 70–100% agreement for the length and helpfulness of the CHAT (*n* = 23). These results were supported by phase 2 qualitative interviews with hospital administrators and practitioners, which revealed the following themes: (1) intuitive and clear design worth sustaining and (2) concerns and proposed solutions for implementation. Findings suggest the CHAT is an appropriate and feasible tool for hospital practitioners to tailor their education and skills training to address care partners’ needs. Identifying care partners’ needs is an important step in ensuring they are prepared to complete their caregiving responsibilities.

## 1. Introduction

Care partners (family members or friends) often assume extensive caregiving responsibilities for our growing older adult population, yet we do not have a reliable method to understand their needs within our healthcare system [1,2]. Research shows the inclusion of care partners into hospital care can improve health outcomes and reduce hospital readmissions and associated costs [3]. Currently, 40 million unpaid care partners of older adults in the US perform a range of hands-on tasks to support older adults before, during, and after hospitalization [4]. Caregiving tasks include, but are not limited to, managing medication, operating medical equipment, assisting with mobility, shopping for groceries, and helping with wound care, dressing, feeding, and toileting [5,6]. Throughout an older adults’ hospitalization and return to home, care partners are additionally responsible for communicating with healthcare practitioners, planning for services and supports, and making decisions about care options in addition to emotional and physical support [7,8]. The US healthcare system depends on care partners to provide these complex care tasks to hospitalized older adults.

To successfully fulfill the caregiving role, care partners rely on education and skills training with healthcare practitioners to better understand and carry out their responsibilities [5,9]. Despite care partners’ integral role, the US healthcare system does not adequately prepare them to provide complex care after hospital discharge. Care partners report dissatisfaction with the education and skills training offered during hospitalization and in turn feel unprepared to meet the needs of older adults [5,8,10]. Care partner unpreparedness negatively impacts the care partner, older adult patients, and the healthcare system. Ill-equipped care partners experience burden, poor health outcomes including physical injury and depression, and emotional and financial stain [10,11,12]. The poor health of some care partners can adversely impact the quality of care and support provided to older adults, which, in turn, can lead to negative health outcomes for the older adult and higher risk of readmission to the hospital [13].

Care partner unpreparedness exists despite partners’ desire to participate in hospital education and skills trainings [14]. Therefore, the barrier to adequately prepare care partners may lie in systems-level hospital processes rather than individual-level care partner motivation. To better support the needs of care partners during their family member or friends’ hospitalization, 44 States and Territories have mandated the CARE (Caregiver Advise, Record, and Enable) Act. The CARE Act requires hospitals to (1) identify and record a patient’s care partner contact information in the medical record, (2) notify the care partner of the patient’s discharge plans, and (3) provide education and skills training on how to care for the patient after discharge [15]. While this policy has established basic standards for the systematic inclusion of care partners in hospitals, research suggests a lack of healthcare systems assessing care partner’s needs during hospitalization [14,16,17]. Furthermore, a recent systematic review found that there are limited assessment tools designed to identify the needs of care partners in the hospital setting [18]. Collectively, these findings reveal a barrier to providing effective education and skills training, which may explain the lack of care partner preparedness for caregiving tasks.

Considering this gap, our team developed the Care Partner Hospital Assessment Tool (CHAT). The CHAT was designed to incorporate a screening–consultative approach. This approach aligns with the screening, brief intervention, and referral to treatment (SBIRT), which is a commonly used public heath technique for delivering preventative interventions [19]. Research shows the SBIRT approach is an effective strategy for instilling change and improving health outcomes across various settings including primary care, emergency departments, and trauma centers [20,21]. Similar to the workflow of SBIRT, CHAT includes a screening phase followed by a brief consultation and educational intervention phase to address care partners’ unmet needs. Accordingly, the CHAT is a structured tool intended to screen for and identify care partners’ needs regarding caregiving preferences and plans and education and skills training. This information is then used to guide clinical decision-making during hospital care.

Despite recent research demonstrating strong content validity of the CHAT, desired implementation outcomes must be assessed before the tool can be administered in a hospital setting. The Consolidated Framework for Implementation Research (CFIR) provides a practical and systematic guide for how to assess potential barriers and facilitators for effective implementation outcomes [21]. Implementation outcomes are defined as the effects of deliberate and purposive actions to implement new treatments, practices, and services [22]. Implementation outcomes should be assessed before clinical outcomes because a tool will not be clinically effective if not implemented well into a system or organization [22].

According to Proctor (2011), appropriateness and feasibility are important implementation outcomes to consider early on in the tool development process. These constructs vary in operational definition; for consistency, appropriateness is defined as “the perceived fit, relevance, or compatibility of an innovation for a given setting; and/or perceived fit of the innovation to address a particular issue or problem”, while feasibility is characterized as “the extent to which an innovation can be successfully used within a given setting” [22]. Different terms that are used for these constructs in the literature include but are not limited to ‘perceived fit’, ‘usefulness’, and ‘utility’ [22].

To examine how well the CHAT can be implemented into a hospital system and identify the needs of care partners of older adults, the primary question of this study was “how appropriate and feasible is the CHAT in identifying care partners’ needs within a hospital setting?” Specifically, the study aimed to describe key stakeholders’ (‘caregiving experts, and healthcare administrators and practitioners’) opinions of the CHAT items and the method for implementing the tool into hospital workflow. With an appropriate and feasible tool that identifies care partners’ needs’, healthcare administrators and practitioners will be better able to provide appropriate education and skills training to care partners in hopes of increasing preparedness to fulfill their caregiving responsibilities at home. (To request a copy of the initial tool, please email the corresponding author).

## 2. Materials and Methods

### 2.1. Study Design

We employed a mixed-methods sequential explanatory design that involved two distinct data collection and analysis phases: (1) quantitative data from a caregiving experts survey and (2) qualitative data from interviews with healthcare administrators and practitioners [23]. In phase 1, the quantitative results from the survey were used to develop a draft of the tool. In phase 2, the qualitative results were used to guide additional revisions to the tool, develop a standard workflow for implementing the tool in a hospital setting, and provide a more complete understanding of the appropriateness and feasibility of the CHAT. Each phase is described in detail below. This exempt study was part of a larger instrumentation study that was approved by the Institutional Review Board at the University of Wisconsin-Madison (UW-Madison) [24].

### 2.2. Quantitative Phase

#### Caregiving Experts

Based on sample size recommendations for instrumentation studies, we purposely selected a panel of at least 10 caregiving experts consisting of care partners of older adults and leading scientists in gerontology, caregiving, and health services research, with an emphasis on discharge planning or home health care [23]. Care partners are considered experts in this study because there is an ethical obligation to involve all who may be affected or benefit from the CHAT [23,25,26]. Care partner eligibility included: (1) provide unpaid care to a family member or friend aged 65 years or older with a chronic illness or disability either before or after hospitalization, (2) be at least 18 years or older, and (3) speak and/or understand English. Scientist eligibility included: (1) have at least five years professional experience in gerontology, caregiving, or health services, (2) be identified by colleagues as a senior scientist in the field, and (3) speak and/or understand English.

### 2.3. Measures

Caregiving experts completed an online survey that contained the initial copy of the CHAT and questions pertaining to the appropriateness, feasibility, relevance, and overall clarity of the CHAT’s content. The CHAT was initially developed based on domains from the Family Caregiver Alliance by the National Center on Caregiving including context, care partner’s perceptions of health and functional status of older adults, care partner values and preferences, well-being of care partner, skills to provide care to older adults, and potential resources that care partners use [24,27]. The study team met monthly in collaboration with the UW-Madison Survey Center to develop and review the survey’s content and logistical flow before distributing. The questions related to appropriateness and feasibility are shown in Table 1. The survey was completed via Qualtrics, a digital survey platform that provides question formats, logic, distribution methods, and data analysis features [28].

### 2.4. Data Collection and Analysis

The survey was distributed electronically via email, which included: (1) purpose of the research study, (2) the right to withdraw at any time, (3) details on risks and compensation, and (4) research team contact information. The survey remained open for six weeks and participants were sent up to three reminder emails to encourage participation. No incentives were provided for completing the survey, and ethics and implied consent were assumed by completion of the survey.

The results of the survey were saved to a secure server in Microsoft Excel. The percent agreement was calculated for questions representing appropriateness and feasibility. Percent agreement was calculated by dividing the total number of participants in agreement by the total number of participant responses. Percent agreement was set at 70% or higher, which has been described as an appropriate threshold for determining consensus in survey research [29]. The percent agreement determined whether participants agreed on the potential length, helpfulness, and disruptiveness of the CHAT within the hospital setting.

### 2.5. Qualitative Phase

#### Healthcare Administrators and Practitioners

Based on sample size recommendations for interviews, we worked with a Clinical Nurse Specialist for Research and Evidence Based Practice to purposely select at least 20 administrators and practitioners from UW Health, a large, academic medical center located in Madison, Wisconsin [30]. UW Health is an integrated health system that serves more than 600,000 patients each year with approximately 1750 physicians and 21,000 staff at seven hospitals and more than 80 outpatient clinics. Eligibility included: (1) at least 1 year of professional experience in geriatric administration or care, (2) work in the hospital setting of UW Health, and (3) speak and/or understand English.

### 2.6. Data Collection and Analysis

Healthcare administrators and practitioners participated in a 60 min, virtual interview via a UW-Madison secure Zoom platform [31]. The interview guide was grounded in and developed based on the caregiving expert survey results and the CFIR [21]. Five questions expanded upon the initial three survey questions addressing appropriateness and feasibility by allowing for open-ended responses related to content, usage, and impact (Table 1). See Appendix A for complete interview guide.

Participants were emailed a Zoom link, a copy of the CHAT, and a link to a demographic survey a week before the interview. Verbal consent was obtained before audio recording. The discussion was led by the principal investigator or graduate research assistant. Participants were compensated with a USD 50 gift card for completion of the interview.

**Figure A1:**
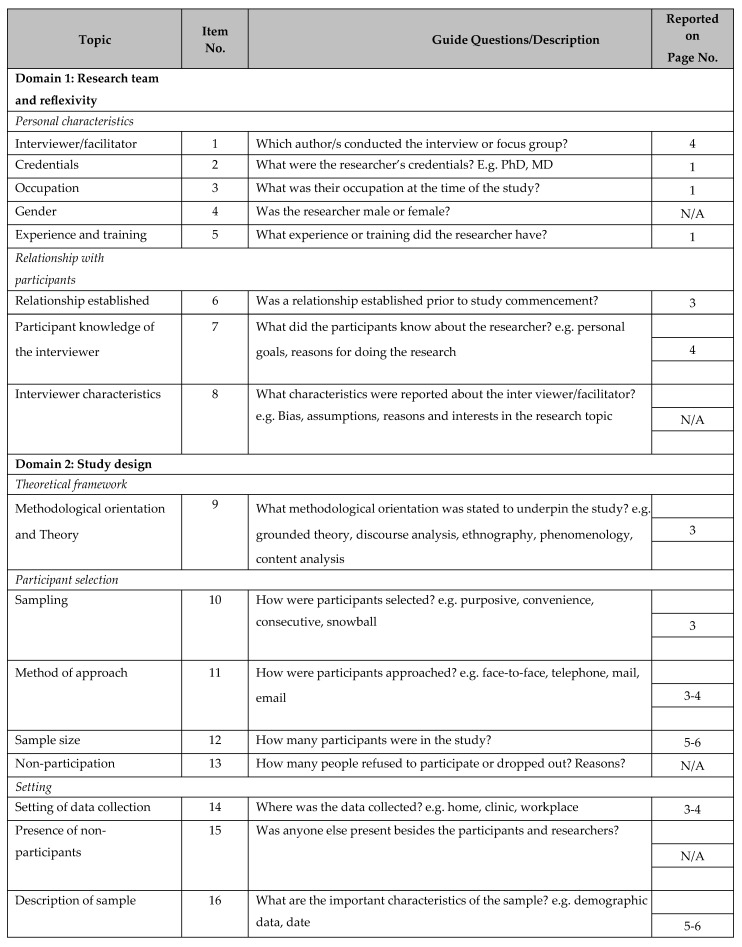

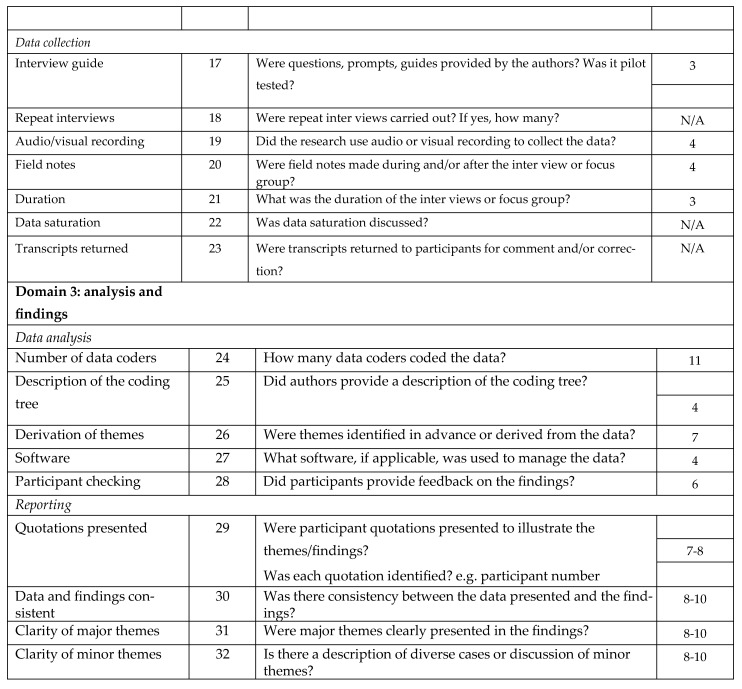
Consolidating criteria for reporting qualitative research (coreq): a 32-item checklist for interviews and focus groups.

Interviews were then de-identified, transcribed, and uploaded to NVivo 12 Pro [32]. The interview data collection and analysis followed the reporting guidelines from “Consolidating Criteria for Reporting Qualitative Research (COREQ): A 32-Item Checklist for Interviews and Focus Groups”, which lists important domains to report for qualitative studies including personal characteristics of the research team, the study design, and data analysis [33]. See Figure A1 in Appendix B for COREQ.

Content analysis was used to describe healthcare administrators’ and practitioners’ opinions of the CHAT in a conceptual form. Content analysis is appropriate to use in explanatory work when there are no previous studies looking at a phenomenon, as is the case with the CHAT [34,35]. The content analysis process was modeled after Elo and Kynäs’s (2008) three-step inductive approach, which included (1) a preparation phase (immersion in the data), (2) an organizing phase (open coding, coding sheets, grouping, categorizing, abstraction), and (3) a reporting phase (models, conceptual systems, maps, or categories). In the preparation phase, the graduate assistant reviewed the interviews and utilized the memo function in NVivo to record personal accounts of thought processes and initial reactions to increase rigor [36]. Throughout the organizing phase, codes and their frequency of occurrence were considered from the researcher’s perspective but also triangulated with other research team members to increase reliability [36,37]. A coding scheme was developed using code functions in NVivo. After reviewing interviews and applying initial codes, analytic memos were written to capture overall impressions and insights about the data collected [37]. In the reporting phase, the content analysis themes and findings were presented to healthcare administrators and practitioners to corroborate themes, foster reflexivity, and further establish trustworthiness [37].

## 3. Results

### 3.1. Quantitative Phase

The survey was distributed to 72 experts and 23 completed the survey. Of the surveys completed, four were care partners and nineteen were leading scientists (see complete demographics in Table 2). The overall response rate was 32%, which is considered average [38]. The percent agreement for the appropriateness and feasibility questions is reported in Table 3.

### 3.2. Qualitative Phase

A total of 21 healthcare administrators and practitioners completed an interview. Participants represented eight different administrative and clinical roles from thirteen different departments within the hospital system. See complete demographic characteristics in Table 4. Two primary themes were identified from the content analysis, including (1) intuitive and clear design worth sustaining and (2) concerns and proposed solutions for implementation.

### 3.3. Intuitive and Clear Design Worth Sustaining

Participants had positive impressions about the content, format, and length of the tool. Words frequently used to describe the content and format of the tool included, “clear”, “logical”, “intuitive”, “accessible”, “pertinent”, and “appropriate”. One practitioner shared, “I don’t think it’s too long compared to other tools we use or have seen” (P1). Participants described the overall tool as valuable and fully supported the feasibility of implementation. A practitioner stated, “we do process improvements all the time- we are open to that change and trying something new” (P2). To increase the buy-in and sustainability, appointing a champion to advocate for and support the use of the tool was advised. Most participants agreed the CHAT should be built into an electronic format for long-term sustainability but supported a paper version for the pilot study. For example, one administrator described, “It could be paper to start, and somebody scans it and attaches it to the patient’s record” and “once built into the electronic system, the information can be routed to those who need the information a little bit more directly” (P6).

### 3.4. Concerns and Proposed Solutions for Implementation

The most prevalent concern reported related to the extensive work demand of administrators and practitioners. Participants worried about the lack of time for additional paperwork, increasing responsibilities for already overwhelmed staff, and limited staffing to effectively implement the CHAT. A practitioner stated, “there’s just people pulled in too many different directions- too much, too many boxes to collect, too many full sheets to fill out, too much.” (P15). To minimize the work demands, participants suggested the CHAT be administered by a non-clinical support staff as soon as possible. Multiple participants suggested that adding the CHAT to the initial admission workflow would be beneficial. Participants recommended that a health unit coordinator analyze the results of the tool and disseminate findings to interprofessional teams via a secure chat, an instant messaging function within the electronic health records. One practitioner stated, “Secure chat is a great idea- it’s taking the place of paging with its instant messaging function and would be a good workflow for the team to easily respond (to the results of the CHAT)” (P15). Team and discharge huddles were advised as optimal opportunities to discuss the results of the CHAT and its implications for education, skills training, and discharge.

Another concern for implementation that most participants identified was the size and complexity of the medical center. One practitioner said, “another barrier is it being such a large hospital, things move really slowly at very large places. It’s so hard to get things going” (P14). Due to the vastness of the organization, participants reported extensive and confusing communication during the discharge planning process. An administrator shared, “specifically to the discharge process, we have a lot of players who are involved in the process and each unit does it differently” (P7). Participants reported that because the CHAT aligned with the mission and values of the medical center, they thought that the challenges associated with size and complexity could be overcome during implementation. All participants shared that they liked CHAT’s strong focus on family-centered care, emphasis on providing effective education to patient and families, and the priority of improving the discharge planning process. Participants were hopeful that the CHAT could assist in quality improvement initiatives related to reducing readmission rates and lengths of stay and increasing care partner satisfaction rates post-hospitalization. Increasing interprofessional communication and streamlining education at transition of care were also frequently cited as positive impacts of the CHAT. For example, one administrator said, “If you involve the care partner and hopefully get the patient the education they need, this could align with a big push that the system is currently thinking about in preventing readmissions- this could go hand in hand” (P17).

A final implementation concern that participants discussed related to the information literacy skills and availability of care partners during a patient’s hospital stay. Participants expressed concern with the low information literacy levels of care partners, which could be a barrier to completing the CHAT. Specifically, some mentioned that if the CHAT was to be delivered electronically, care partners may need more assistance with using the technology. For example, a practitioner said, “they (care partners) aren’t tech savvy, they don’t like the iPad or electronics surveys” (P15). Other participants described how care partners may be limited in their visitation hours due to the COVID-19 pandemic or commonly come to visit after work hours, making it difficult for hospital staff to connect in-person with them. Another practitioner stated, “We work during the day, if their family member works during the day, we don’t get to see them” (P7). To combat these concerns, various participants emphasized the importance of interprofessional, collaborative practice with the care partner as part of the team. Utilizing telephone calls or other virtual platforms to provide education and skills training could alleviate availability challenges by offering more flexible communication options. For example, a practitioner agreed, “I wonder if we could use video and video visits better with people that aren’t allowed to have visitors or it may not be the caregiver who’s going to be doing that dressing change, so can we utilize technology to be able to bring the two together” (P5).

## 4. Discussion

We evaluated the appropriateness and the feasibility of the CHAT for use with care partners of older adults in the hospital setting. We found that caregiving experts agreed that the CHAT was an appropriate length, could be helpful in identifying care partner’s needs, and would not be disruptive to patient care. Interviews with healthcare administrators and practitioners further supported the experts’ opinions of the tool and provided additional insight into how the CHAT could be implemented, sustained, and impactful in hospital care. The connected results from both the quantitative and qualitative phases indicate the CHAT is an appropriate and feasible assessment tool for a hospital system that could ensure care partners are prepared to fulfill their caregiving responsibilities.

**Figure 1:**
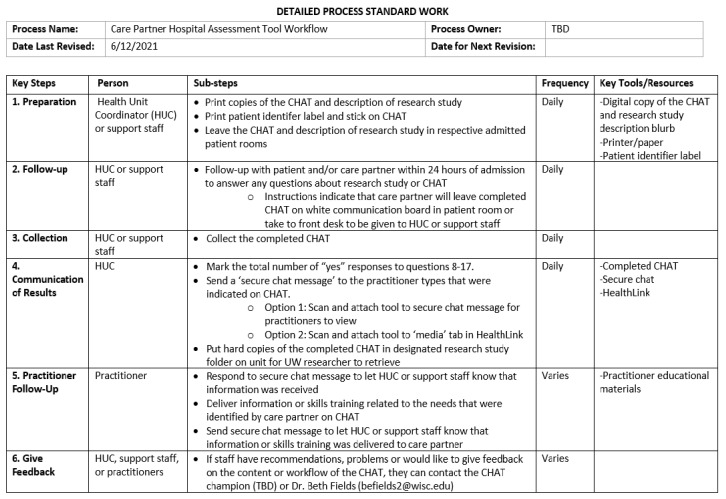
Example workflow for CHAT implementation at UW Health.

The CHAT could be implemented into routine practice with limited changes to the medical center system. Research supports the use of available resources, roles and responsibilities, and adequate training for successfully incorporating new initiatives into practice [39,40,41]. The CHAT implementation process will make use of current employees, workflows, and resources for the administration, interpretation, and training related to the tool. For example, current non-clinical support staff could administer the tool at an established admission, and the training for the CHAT could occur during scheduled team meetings. See Figure 1 for proposed CHAT workflow. Intertwining CHAT processes will help ensure uptake within the system without overburdening staff.

Identifying a champion, or multiple champions, could ensure the CHAT is implemented and sustained within a hospital system. A champion is a staff person within an organization who has an intrinsic commitment to implementing change and works diligently to move an innovation forward [42]. The medical center frequently relies on champions to increase buy-in and uptake of new initiatives within the system. Historically, champions have been vital in integrating change into routine, promoting evidence-based practice, and bolstering positivity [42,43]. Many of the participants, specifically directors/managers, were in favor of identifying at least one champion to promote the CHAT and serve as a front-line expert in the use of the tool.

The CHAT tool has the potential to increase communication among healthcare administrators, practitioners, and care partners, which may have system-wide impacts. Studies have found that lack of communication around discharge led to poor patient outcomes and suggested that interprofessional communication be standardized [44]. Furthermore, a recent study found that interprofessional communication improves discharge decision making for hospitalized patients [45]. Participants overwhelmingly reported that the CHAT could increase and streamline interprofessional communication, especially at discharge, by providing a team a standardized tool for discussion. Findings from the CHAT could also facilitate discussion between hospital staff and the care partner, which has been proven to reduce readmission rates and malpractice suits and increase family satisfaction [3,46]. The increased communication related to the CHAT could also contribute to its sustainability if positive outcomes are shared with hospital staff.

### Strength and Limitations

Several strengths of this study enhanced the work. Triangulation of interview codes was performed with research team members to decrease risk of bias and increase dependability of the results. The study design involved community-based stakeholders, including care partners and administrators and practitioners at the medical center who will ultimately be affected by the implementation of the CHAT. Lastly, results from this study will contribute to a limited body of literature on assessment tools for care partners of hospitalized older adults.

Several limitations from this study warrant specification. Administrators and practitioners from the qualitative phase represented only one health system, which limits generalizability to other systems. Although interviews allowed time for additional comments, certain opinions from key stakeholders may have been omitted, such as admissions and non-clinical support staff.

## 5. Conclusions

The CHAT is a promising, appropriate, and feasible assessment tool to identify the needs of care partners of hospitalized older adults. Caregiving experts and hospital administrators and practitioners valued the content, design, and purpose of the tool. Our findings indicate methods for how the CHAT could be implemented into hospital practice, potentially increasing interprofessional communication, and patient and family satisfaction. Future research should evaluate the effectiveness of the CHAT in a hospital setting to determine its impact on readmission rates, as well as care partner preparedness and satisfaction.

### Clinical Implications

- Healthcare systems could implement CHAT without overburdening practitioners and staff.
- The CHAT could equip care partners to fulfill caregiving responsibilities at home by identifying and triggering appropriate hospital-based education and skills training.
- The CHAT could facilitate interprofessional communication around hospital discharge.

## Figures and Tables

**Table 1:** Example healthcare administrator and practitioner interview questions.

1. What are some things you like about the CHAT? (length, content, appearance)
2. What are some things you dislike about the CHAT? (length, content, appearance)
3. Is there a strong need for the CHAT? Why or why not?
4. How do you think the CHAT could impact your practice?
5. How do you think the CHAT could impact your organization?

**Table 2:** Demographic characteristics of caregiving experts (*n* = 23).

Variable	Frequency (%)
Female	15 (65)
*Expert affiliation*	
Academic *	6 (32)
Academic medical *	9 (47)
Industry *	2 (11)
Government *	2 (11)
*Location in USA*	
Northeast	14 (61)
Southeast	3 (13)
Southwest	1 (4)
West	1 (4)
Midwest	4 (17)

* Academic = professor, faculty at an academic institution; Academic medical, faculty at an academic institution and practicing clinician; Industry = position in a non-government agency related to geriatrics; Government = position in a government agency related to geriatrics.

**Table 3:** Survey questions addressing appropriateness and feasibility and percent agreement.

Question	Response Options	Number in Agreement (%)
1. For the hospital setting, the CHAT is:	The right length	17/23 (73)
	Too long/short	
2. Do you think the CHAT will be helpful in identifying care partners’ needs during hospital care?	Yes	16/23 (70)
	No	
3. Do you think the CHAT will be disruptive to the delivery of care to the patient?	Yes	
	No	23/23 (100)

**Table 4:** Demographic characteristics of healthcare administrators and practitioners (*n* = 21).

Variable	Frequency (%)
Female	18 (85)
White	21 (100)
Hispanic or Latinx	1 (5)
*Years in Geriatric Care*	
0–4 years	8 (38)
5–10 years	2 (10)
11–19 years	7 (33)
20+ years	4 (19)
*Role*	
Director/manager	7 (33)
Physical Therapist	4 (19)
Nurse	4 (19)
Occupational Therapist	3 (14)
Clinical Nurse Specialist	2 (10)
Pharmacist	2 (10)
Hospitalist	1 (5)
Speech/Lang. Pathologist	1 (5)
Dietician	1 (5)
*Department*	
General Medicine Inpatient	5 (24)
Rehabilitation Therapy	3 (14)
Acute Care for the Elderly	2 (10)
Coordinated Care	2 (10)
Center for Clinical Knowledge Management	
Knowledge Management	1 (5)
Health Information	
Health Information Management	1 (5)
Informatic Services	1 (5)
Nursing Informatics	1 (5)
Nursing Research &	
Nursing Research & Evidence	1 (5)
Nutrition	1 (5)
Patient Experience	1 (5)
Pharmacy	1 (5)
Transitional Care Group	1 (5)

## Data Availability

The data presented in this study are available on request from the corresponding author.

## Appendix A

### Phase 2 Interview Guide

Could you share with us your current roles or positions within *?

**I’d like to start by learning your initial impressions of CHAT**.

1. What are some of the things you like about CHAT?
  - Content, appearance, clarity of instructions?
2. Are there things you don’t like about CHAT?
  - Are there any suggestions to adapt/improve the content or delivery of CHAT?
3. Is there a strong need for CHAT? Why or why not?
4. Do you think CHAT will be effective in addressing unmet information and training needs of care partners of hospitalized adults?
  **I’d like to talk about the UW Health network and culture**.
5. Are meetings, such as staff meetings, held regularly?
  - Do you typically attend? Who?
  - What proportion of staff typically attend?
  - How often are the meetings held?
  - What is a typical agenda? How helpful are these meetings?
6. How do you typically find out about new information, such as new initiatives, accomplishments, issues, new staff, staff departures?
7. How do you think UW Health’s culture will affect the potential implementation of CHAT?
  - To what extent are new ideas embraced and used to make improvements?
  **I’d like to discuss ways to implementing/sustaining CHAT**.
8. How would you envision UW Health implementing CHAT?
  - What special arrangements would you imagine needing to do to implement CHAT?
    - Leadership engagement, approvals?
    - Do you think UW Health will be able to make these changes? Why or why not?
  - Which healthcare practitioner(s) do you think should administer CHAT?
  - Which healthcare practitioner(s) should follow-up with care partners to provide information or training related to specific items of the Skills & Support section of CHAT?
  - How would UW Health prepare practitioner(s) to deliver CHAT?
    - How confident are you that UW Health would be able to successfully implement CHAT?
  - What challenges would you expect from implementing CHAT?
  - Are there any suggestions to address these challenges?
  - Who will decide whether changes are needed to CHAT?
9. How does CHAT compare to other alternatives that may have been considered to address care partner’s needs?
10. How do you think CHAT could impact your own practice?
11. How do you think CHAT could impact *?
